# Functional Layered Double Hydroxide Nanohybrids for Biomedical Imaging

**DOI:** 10.3390/nano9101404

**Published:** 2019-10-02

**Authors:** Wenji Jin, Dae-Hwan Park

**Affiliations:** 1 Department of Nano Materials Science and Engineering, Kyungnam University, Changwon, Gyeongsangnamdo 51767, Korea; jinwenji7@163.com; 2 College of Chemistry and Environmental Engineering, Jiujiang University, Jiujiang 332005, China

**Keywords:** layered double hydroxide, nanoparticle, nanohybrid, bio-imaging, therapy

## Abstract

Biomedical investigations using layered double hydroxide (LDH) nanoparticles have attracted tremendous attentions due to their advantages such as biocompatibility, variable-chemical compositions, anion-exchange capacity, host–guest interactions, and crystallization-dissolution characters. Bio-imaging becomes more and more important since it allows theranostics to combine therapy and diagnosis, which is a concept of next-generation medicine. Based on the unique features mentioned above, LDHs create novel opportunities for bio-imaging and simultaneous therapy with LDHs-based nanohybrids. This review aims to explore the recent advances in multifunctional LDH nanohybrids ranging from synthesis to practical applications for various bio-imaging with therapeutic functions. Furthermore, their potential both as diagnostic agents and drug delivery carriers will be discussed with the improvement in noninvasive bio-imaging techniques.

## 1. Introduction

Layered double hydroxides (LDHs) have been extensively explored in the nano-bio fields based on their inherent two-dimensional (2D) layered structure, excellent biocompatibility and biodegradability [1,2,3,4,5,6,7]. Since intercalative nanohybrids of DNA in LDH was proposed as “bio-inorganic nanohybrids” for the first time in 1999 [8], LDHs have received continuous attentions in biomedical applications with multifunctional nanohybrid systems. A representative research is related to nano contrast agent systems that are essential for the development of bio-imaging technology [9,10,11,12]. Successful manipulation on the nanometer scale of LDH can provide novel perspectives in the design of multifunctional nanohybrids, which open up a promising field for applications in bio-imaging with simultaneously effectual therapeutic functions.

**Figure 1:**
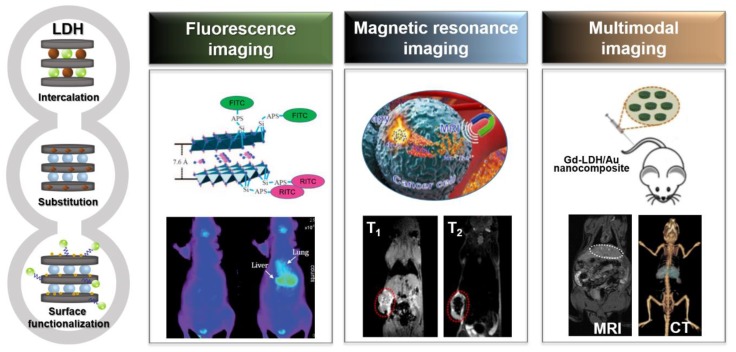
Overview of functional layered double hydroxide nanohybrids (left) and their related application systems (right): fluorescence imaging, reproduced from [20,35], with permission from the Royal Society of Chemistry, 2012 [20] and Wiley-VCH Verlag GmbH & Co. KGaA, 2009 [35], magnetic resonance imaging (MRI), reproduced from [11,12], with permission from the Royal Society of Chemistry, 2017 [11] and American Chemical Society, 2019 [12], and multimodal imaging, reproduced from [10], with permission from Elsevier Ltd., 2013.

Bio-imaging techniques with high resolution and sensitivity aid in both the detection of diseases and the understanding of biological phenomena. It is rapidly evolving through the convergence of advanced imaging technology, medicine, molecular biology, genetics, cytology, nuclear medicine, chemistry, pharmacology, physics, etc. It is also the latest multidisciplinary field that contributes to the diagnosis, treatment and prevention of disease as well as continuous monitoring of post-treatment condition [13,14,15,16,17,18]. Three-dimensional (3D) bioprinting, a combination of 3D printing techniques and medical technology, is one core technology of the 4th industrial revolution era [19]. 3D bioprinting has expanded widely in patient-customized applications ranging from production of artificial skin, organs, and even to blood vessels, but in order to pursue clinical efficacy, it is essential that 3D bioprinting is based on fast, reliable, and high-quality data through bio-imaging. The representative bio-imaging modalities in either clinical settings or preclinical research are optical fluorescence imaging [6,9,20,21,22,23,24,25], magnetic resonance imaging (MRI) [26,27,28,29] and multimodal imaging [30,31,32,33,34], which has been utilized via the combination of different imaging methods with various image performances, such as sensitivity, resolution and accuracy (Figure 1. right).

In order to achieve highly accurate images for early diagnosis and effective treatment, continuous trials in performance enhancements of noninvasive bio-imaging techniques are in progress. One strategy is the use of advanced contrast agents to overcome instrumental limitations including resolution or sensitivity of a particular imaging technique to enhance the accuracy of bio-imaging [35,36,37,38,39]. Another method under study is multimodality imaging, which is a synergistic integration of various modalities to complement the weaknesses with the strengths of the other imaging modalities [40,41,42,43,44,45].

In particular, LDHs have been serving as a suitable material in the development of nano contrast agents due to their tunable properties based on unique 2D crystal structure as well as internal and external surface properties [46,47,48,49,50,51,52,53,54,55,56]. (a) The negatively charged contrast agent can be readily immobilized in the interlayer space of LDHs and stabilized mainly by an electrostatic attraction, whereby a stable nano contrast agent with 2D layered structure can be produced. (b) LDH nanoparticle-based contrast agents can be produced by doping various metal ions (Gd^3+^, Mn^2+^, etc.) through a suitable lattice manipulation within the positively charged metal hydroxide layer. (c) The surface functionalization of the LDH nanoparticles, usually done by introducing photofunctional components, such as fluorescent probes or gold nanoparticles, can be achieved as a nano contrast agent material (Figure 1. left). In addition, because of high loading capacity, protection of drugs and genes within interlayer galleys, and sustained release with pH-controlled solubility properties, LDHs have been thus widely researched as bio-imaging agents and drug delivery carriers simultaneously with both diagnostic and therapeutic functions [57,58,59,60,61].

We focus here on recent developments in the field of nanoscale contrast agent materials for enhanced bio-imaging application based on the multifunctional LDHs. This review presents an overview of LDH nanohybrids along with their various synthetic strategies and potential applications for some representative bio-imaging modalities with therapeutic functions. The emerging challenges in related research will be highlighted with in vitro and in vivo studies.

## 2. Design of Functional LDH Nanohybrids for Biomedical Imaging

As one of the 2D layered materials, LDHs consist of positively charged metal hydroxide layers and the anions in the interlayer space in order to satisfy the charge neutrality condition. The general formula of synthetic LDHs is [M^2+^_1−x_M^3+^_x_(OH)_2_]^x+^(A^m−^)_x/m_∙nH_2_O, where M^2+^/M^3+^ represents a divalent/trivalent metal cation respectively, and A^m−^ is a negatively charged molecule. To understand soft chemistry of LDHs, it is required to describe the structure of brucite mineral Mg(OH)_2_, where the Mg^2+^ ion is coordinated with six OH^−^ ligands to form an octahedron crystal structure, and formed octahedrons are bound to another by sharing edges to build up infinite sheets, which are then stacked layer by layer to achieve lamellar structure by strong hydrogen bonding. In the LDH structure, metal divalent cations, such as Mg^2+^, Fe^2+^, Zn^2+^, Cu^2+^, Ni^2+^, Co^2+^, and Ca^2+^ are partially substituted by trivalent cations, such as Al^3+^, Gd^3+^, Fe^3+^, Co^3+^, and Ga^3+^, so that the positively charged host layer can be obtained. To compensate the positive charge in LDH lattice, anionic species should be stabilized in the interlayer space, which can be further exchanged with other ones—atomic ions, molecular clusters, and even supramolecular anions [2].

Various kinds of organic or inorganic anions have been introduced between the metal hydroxide layers to prepare functional nanohybrids based on LDHs [62,63]. There are several lattice engineering routes for hybridization such as co-precipitation, ion-exchange, calcination–reconstruction, and exfoliation–reassembling strategies. Such intercalative nanohybrids could be formed by many different chemical driving forces—mainly electrostatic interaction, partly coordinative bonding, and molecular interactions including hydrogen bonding and van der Waals interaction at interfaces between LDH host planes and guest species.

**Figure 2:**
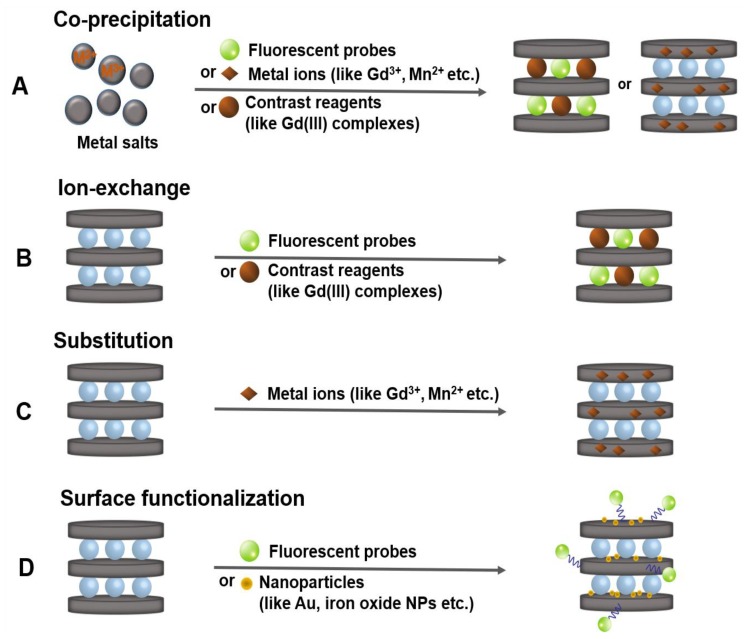
Synthesis strategies of functional LDH nanohybrids for bio-imaging, (**A**) coprecipitation, (**B**) ion exchange, (**C**) substitution, and (**D**) surface functionalization.

As described in Section 1, the structure of functional LDH nanohybrids for bio-imaging application is largely composed of three types: an intercalation type, in which fluorescent substance or a typical example of Gd(III) complexes, diethylenetriamine pentaacetic acid (Gd-DTPA) is intercalated into the guest layer between inorganic materials; a substitution type doping various metal ions such as Gd^3+^ and Mn^2+^ in the metal hydroxide host layer; or a surface functionalization type through surface chemistry using fluorescent substance or gold nanoparticles. The nano contrast agent materials with described structures are primarily produced through the following four synthesis strategies reported so far [5,7,46]. (1) The co-precipitation is a facile and economic method of preparing bio-hybrids (Figure 2A). In this reaction, an aqueous solution of mixed two or more metal ions including Gd^3+^ or Mn^2+^, which can improve MRI performance, is immediately precipitated by base titration to form nanoscale imaging agent. Negative charged imaging functionalized molecules can simultaneously be intercalated into layered LDHs. (2) The most widely used alternative post-synthetic intercalative method to prepare LDHs-supported imaging contrast agent has been developed by ion-exchange reaction (Figure 2B). In this process, the pristine LDHs are prepared with exchangeable anions first, and then the contrast-enhancing agents, e.g., Gd-DTPA, can replace the pre-occupied ones within interlayer space of LDHs matrix. (3) The substitution of metal ions (like Mn^2+^, Dy^3+^, Cu^2+^, etc.) into LDH lattices is another suitable way to develop LDHs-based functional nanomaterials (Figure 2C). For instance, the synthesis of Mn^2+^ doped LDH nanoparticles can be conducted by two steps: first co-precipitation to get the pristine LDHs and then subsequent isomorphic substitution can occur to form Mn-LDH with evenly dispersed Mn^2+^ ions on the host layers. (4) The surface functionalization of the LDH nanoparticles is possible by using the OH groups around metal ions and can be utilized as a nano contrast agent material by introducing photofunctional components such as fluorescent substances or gold nanoparticles (Figure 2D).

Similarly, the anticancer drugs, biomolecules (like DNA, siRNA) and other therapeutic agents can be introduced into LDHs through lattice engineering strategies like intercalation, surface functionalization, etc. LDHs can provide efficient protection for biological functional molecules to enhance their stability, as well as bioavailability due to anion exchange capacity and high host layer charge density. They have been thus widely researched as controlled-release and target-oriented drug delivery system [3,4].

## 3. LDH Nanohybrids for Bio-Imaging Applications with Therapeutic Functions

During the last decade, continuous attempts have been made in developing LDHs for novel bio-imaging applications with therapeutic functions. In this section, we will highlight some recent experimental findings in both in vitro and in vivo studies on fuorescence imaging, MRI, and multimodal imaging. Their possible applications as nanocontainers for therapeutic agents including genes, anti-cancer drugs, and photothermal reagents will also be discussed along with the bio-imaging functions.

In addition to the synergistic combination of different imaging modalities, the drug or gene molecules can be intercalated into the interlayer or onto the surface of the nanoparticle probes through bioconjugation chemistry, producing theranostic agents. There are some studies on LDH-based nanohybrids for multimodal imaging as well as desirable combination therapies, which use more than one medication or modality to realize synergistic treatment. LDHs have been extensively studied as a theranostic delivery carrier for a variety of therapies, including chemo-, gene-, photo-, boron neutron capture-, and even immunotherapy [64,65,66,67,68,69,70,71,72,73,74,75,76,77,78].

### 3.1. Fluorescence Imaging

Fluorescence imaging techniques play an important role in the bio-imaging owing to their advantage of obtaining high spatial and temporal resolution with good sensitivity as well as the practical utility in real-time monitoring caused by their relatively short acquisition time. It has developed rapidly with the development of nanotechnology which has led to the bio-imaging at subcellular or molecular level [41,42].

#### 3.1.1. LDH Nanohybrids for Fluorescence Imaging

In 2000, Choy’s group reported that the stable bio-LDH hybrids could be introduced into cells by intercalating negatively charged biomolecules DNA into the interlayer of layered LDHs used as nonviral vectors [22]. The bio-LDH hybrids had been achieved by simple ion-exchange reaction and the DNA was efficiently protected by LDHs. The charge neutralization weakened the electrostatic interactions between the anionic biomolecule and the negatively charged cell membrane, enhancing the transfer of the DNA-LDH hybrids into the cell through endocytosic means. In this study, the cellular uptake behaviors were demonstrated through the fluorescence imaging by intercalating a green fluorescent dye, fluorescein isothiocyanate (FITC) between the LDH layers. Once within the cells, the encapsulated LDH slowly dissolved in the lysosome exhibiting slight acidity (pH = 4–5), and thereby the intercalated biomaterial was released.

In 2006, in vitro studies with FITC-LDH were performed to demonstrate the possibility of internalization of LDH nanoparticles into cells principally through the clathrin-mediated endocytosis [9]. However, it has been noticed that the mechanism of selectively permeating into cell is effective only at LDH nanoparticle size of 300 nm or less. And it was also showed that the internalization of LDHs loaded with an anticancer drug methotrexate (MTX) via clathrin-mediated endocytosis allowed the delivery of MTX-LDH into cells and, thus, delivery efficiency was effectively improved.

Related researches have been continuously conducted, and in 2012 Choy et al. presented the intracellular trafficking pathway and mechanism of FITC-labeled LDH nanoparticles of different sizes (50 nm, 100 nm) [21]. It has been proved that the nanoparticle size plays an important role in cellular uptake and intracellular trafficking of drug or gene encapsulated in LDH nanoparticles and it can maximize therapeutic effect by controlling the particle size. Especially, 100 nm LDH nanoparticles are more effective in gene delivery because they could effectively escape and avoid endolysosomal degradation.

#### 3.1.2. LDH Nanohybrids for Fluorescence Imaging with Therapeutic Functions

**Figure 3:**
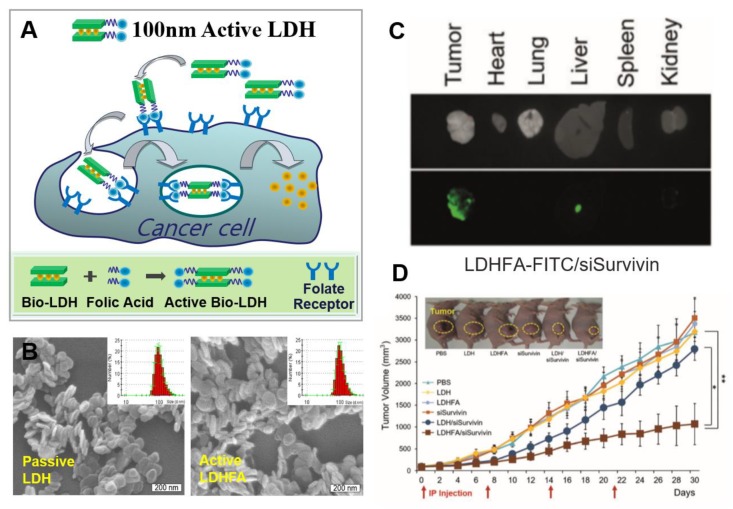
(**A**) Schematic illustration of intracellular trafficking pathway and synthetic procedure of active bio-LDHFA nanoparticles, (**B**) SEM images and size distributions of passive LDH and active LDHFA, (**C**) ex vivo biodistribution of the LDHFA-FITC-siSurvivin in the xenograft model: optic (top) and fluorescence (bottom) images, (**D**) in vivo anti-tumor efficacy of KB tumor-bearing mice treated for 30 days with each sample via intraperitoneal injection once every seven days (inset photo image shows the mice on 18 days post-treatment), reproduced from [6], with permission from Wiley-VCH Verlag GmbH & Co. KGaA, 2016.

More recently, Choy et al. reported the inhibition of tumor growth utilizing LDH nanovectors conjugated with specific targeting ligand folic acid (FA) and loaded with Survivin siRNA (siSurvivin). [6] They described in vitro and in vivo delivery system with two groups of LDH-siSurvivin nanohybrids, one being the LDH-siSurvivin hybrids for passive delivery based upon the permeability and retention (EPR) effect and enhanced clathrin-mediated endocytosis and the other being LDHFA-siSurvivin hybrids for folate receptor (FR)-mediated active targeting (Figure 3A). The negatively charged siSurvivin was self-assembled with LDH or LDHFA by electrostatic interaction. Both LDH-siSurvivin and LDHFA-siSurvivin nanohybrids were formed as uniform hexagonal shaped platelets with a size of 100 nm (Figure 3B), which is proper to avoid endolysosomal degradation. With fluorophore labelling, LDH-FITC-siSurvivin and LDHFA-FITC-siSurvivin were prepared and used for ex vivo biodistribution and in vivo anti-tumor effect. At 30 min after intraperitoneal injection, both groups showed greater tumor selectivity when compared to other organs, including lung, liver, and spleen. The fluorescence intensity of the group injected with the LDHFA-FITC-siSurvivin hybrids was found to be 1.2-fold higher (Figure 3C), and it was confirmed that the FA ligands improved the transmission efficiency. It was also indicated that LDHFA-siSurvivin gene delivery system using LDHFA active nanovector improved the inhibition rate of tumor growth 3.0-fold after 30 days of intraperitoneal injection in KB tumors compared to the LDH-siSurvivin comparison group (Figure 3D).

**Figure 4:**
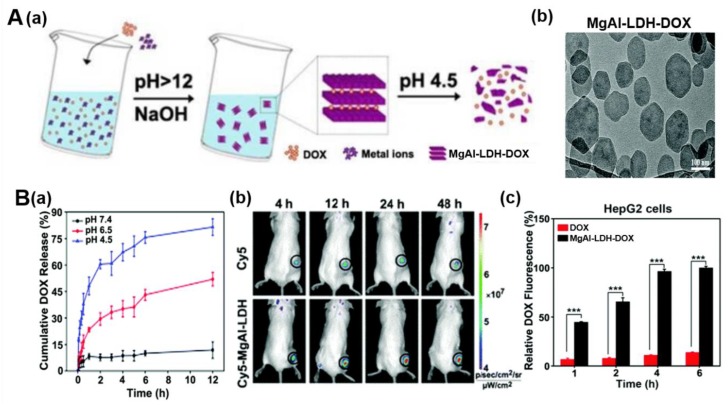
(**A**) (**a**) Schematic illustration of synthetic procedure and pH-responsive DOX release, (**b**) TEM image of MgAl-LDH-DOX, (**B**) (**a**) pH-responsive DOX release profiles from MgAl-LDH-DOX in PBS at 37 °C, (**b**) in vivo fluorescence images of H22 tumor-bearing mice at different time courses after intravenous injection of free Cy5 or Cy5-MgAl-LDH at the Cy5 dose of 0.3 mg/kg, respectively, (**c**) intracellular DOX content in HepG2 cells treated with free DOX or MgAl-LDH-DOX at DOX concentration of 3 μg/mL for different time intervals by flow cytometry, reproduced from [23], with permission from the Royal Society of Chemistry, 2018.

Yang et al. developed doxorubicin (DOX)-loaded MgAl-LDH nanohybrids for efficient cancer therapy (Figure 4) [23]. DOX is a kind of widely used DNA-targeting anticancer drug with positive charge. However, its clinical use is limited because it causes damage to the heart. MgAl-LDH-DOX was obtained through a facile strategy in a base-catalyzed co-precipitation reaction (Figure 4A(a)). During this reaction, Mg^2+^ and Al^3+^ ions in the LDH host layer might be bound to the NH_2_ of DOX through coordination interaction under alkaline condition. Both MgAl-LDH and MgAl-LDH-DOX showed well-defined plate morphology with the particle size of 180 and 220 nm, respectively (Figure 4A(b)). They also demonstrated that MgAl-LDH-DOX nanohybrids showed effective pH-responsive drug release in tumor acidic microenvironment (Figure 4B(a)). The in vivo biodistribution of nanoparticles, which plays an important role for biological activity, was investigated through fluorescence imaging. The Cy5 fluorescence intensity increased over time in tumors both of Cy5-conjugated MgAl-LDH- and free Cy5-treated groups. However, Cy5-conjugated MgAl-LDH-treated groups showed much stronger fluorescence intensity (Figure 4B(b)). Furthermore, more DOX was detected in tumors of H22 tumor-bearing mice compared with free DOX. These results revealed that MgAl-LDH-DOX exhibits good tumor targeting capacity and enhanced cellular uptake. As in the result of in vitro study in human hepatocarcinoma HepG2 cells, the intracellular DOX fluorescence intensity was stronger in MgAl-LDH-DOX-treated group than free DOX-treated group, namely MgAl-LDH-DOX exhibited 7-fold more internalization into HepG2 cells after 6 h treatment (Figure 4B(c)). Correspondingly, enhanced treatment effect was shown in MgAl-LDH-DOX-treated group by detecting stronger inhibition in cell viability against HepG2 cells. In vivo study in H22 tumor-bearing mice confirmed the excellent anticancer activity of MgAl-LDH-DOX with decreased heart toxicity. It exhibited an inhibition rate in tumor growth of 64%, while free DOX with 36% inhibition rate.

### 3.2. Magnetic Resonance Imaging (MRI)

MRI holds the advantages of being a noninvasive technique for high-resolution anatomical imaging and 3D tomography, for which it is now one of the most powerful biomedical imaging modalities. MRI is able to measure the proton relaxation processes of water in tissues and can record such relaxation processes to reconstruct as 3D gray scale images [38,44]. To enhance the contrast, contrast agents are used to shorten the T_1_ (longitudinal relaxation time) and T_2_ (transverse relaxation time) of water. According to such two different relaxation pathways, obtained images can be classified as T_1_-weighted or T_2_-weighted images. T_1_ contrast agents are used to enhance longitudinal relaxation processes, producing brighter signals by using a paramagnetic material such as Gd^3+^ or Mn^2+^ metal ion [10,11,12,79] to shorten the T_1_. On the other hand, T_2_ contrast agents are made of a superparamagnetic material such as iron oxide nanoparticles [12,38,80,81], which can enhance the transverse relaxation processes by shortening T_2_ and thus result in darker signals. Studies are underway on advanced contrast agents that can boost up imaging sensitivity through the contrast enhancement in regions of interest in T_1_ or T_2_ images.

#### 3.2.1. LDH Nanohybrids for MRI

A representative example of pioneering studies of the MRI contrast agent based on LDHs is to intercalate a contrast agent such as Gd-DTPA into the interlayer of LDHs to form the T_1_-weighted contrast agent system [27,29]. The synthesis of nanohybrids is possible by the co-precipitation or ion-exchange after the production of the LDH. Since then, many researchers have continued to develop T_1_-weighted contrast agent by doping divalent or trivalent metal ions (Gd^3+^, Dy^3+^, Mn^2+^, Cu^2+^, etc.), available as contrast agents, onto the LDH host layers [10,11,26,28,56]. The therapeutic functions including drug delivery and gene therapy along with imaging functions were also widely investigated. Most recently, the concurrently enhanced T_1_/T_2_-weighted contrast agent was reported and has attracted special attention for clinical applications in MRI with improved accuracy [12].

**Figure 5:**
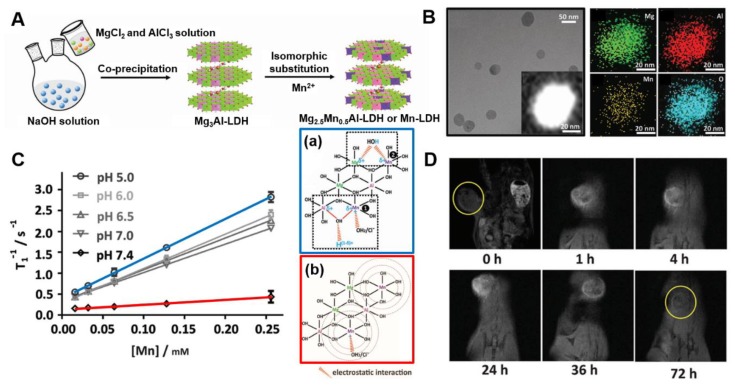
(**A**) Schematic illustration of synthetic procedure, (**B**) TEM image, STEM image and the corresponding element mapping of Mn-LDH nanoparticles, (**C**) Plot of T-1 versus Mn concentration of Mn-LDH nanoparticles after co-incubation with different pH buffer solution at 37 °C for 4 h and 2D atomic structure models of Mn-LDH dispersed in pH 5.0 (**a**) and pH 7.4 (**b**) buffer, (**D**) in vivo MR imaging in the melanoma tumor-bearing mouse after intravenous injection of BSA/Mn-LDH nanomaterial within 72 h, from [26], with permission from WILEY-VCH Verlag GmbH & Co. KGaA, 2017.

Xu et al. have developed a novel Mn-LDH T_1_-MRI contrast agent by isomorphic substitution, replacing partial Mg^2+^ with Mn^2+^ metal ions in Mg_3_Al-LDH (Figure 5A) [26]. The devised nano contrast agent showed satisfactory pH-ultrasensitive T_1_ relaxivity even at very weakly acidic environment with pH 6.5–7.0, i.e., the range of tumor microenvironment. It showed superb T_1_ relaxivity in pH 5.0–7.0 (9.48 mM^−1^s^−1^, 6.82 mM^−1^s^−1^ at pH 5.0, 7.0, respectively, vs. 1.16 mM^−1^s^−1^ at pH 7.4) The Mn-LDH can be efficiently internalized into tumor cells and can effectively acquire MRI images over a period of as long as 2 days or more in vivo (vs. conventional Gd-based contrast agents with less than 2 h). It is a remarkable T_1_ contrast with high relaxivity, ultrasensitive pH response, and prolonged imaging time, widening its potential application with both diagnostic and therapeutic functions.

The Mn-LDH exhibits extremely sensitive properties to pH, which may be due to the microstructure change of Mn^2+^ in the LDH host layer and is demonstrated by extended X-ray absorption fine structure (Figure 5C). The in vivo T_1_-MRI performance was demonstrated with bovine serum albumin (BSA)/Mn-LDH pre-coated with BSA to prevent aggregation of nanoparticles in vivo. BSA/Mn-LDH are produced with a size of ≈68 nm, and it was demonstrated that there was no distinct change in the crystal structure and pH ultrasensitivity for MR imaging before and after coating. As shown in Figure 5D, a remarkable T_1_-enhanced signal was clearly observed in the tumor area 1 h after intravenous injection of BSA/Mn-LDH nanoparticles, and the intensity continuously increased for 24 h probably due to the ERP effect. The prolonged MR imaging time compared to these of conventional Gd-based contrast agents is expected to provide an alternative to in vivo disease therapy monitoring, such as anticancer and regenerative medicine. Additionally, the cytotoxicity test for Mn-LDH nanoparticles was found to exhibit low cytotoxicity compared to the conventional Gd-based contrast agent.

**Figure 6:**
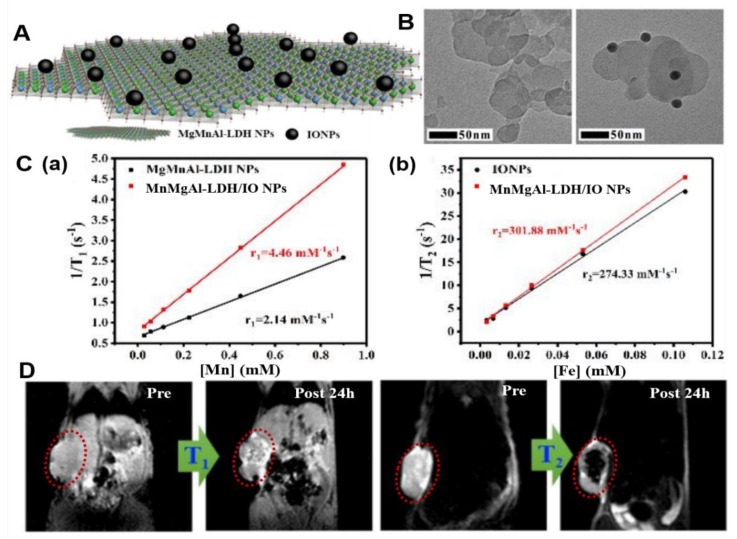
(**A**) Schematic illustration of MnMgAl-LDH/IO NPs, (**B**) TEM images of MnMgAl-LDH (left) and MnMgAl-LDH/IO NPs (right), (**C**) analysis of relaxation rate 1/T_1_ vs. Mn (**a**) and 1/T_2_ vs. Fe (**b**) concentration for contrast agents at pH 6.5, (**D**) T_1_-and T_2_-weighted MR images of breast tumor bearing mice before and after intratumoral injection of BSA/MnMgAl-LDH/IO NPs, reproduced from [12], with permission from American Chemical Society, 2019.

Most recently in 2019, a novel MnMgAl-LDH/iron oxide nanoparticle (MnMgAl-LDH/IO NP) has been successfully achieved as a concurrently enhanced T_1_ and T_2_-weighted MRI contrast agent for accurate tumor diagnosis with sensitive pH response (Figure 6) [12]. In this study, which purposed at eliminating the interference effect and enhancing the selectivity, the well-crystallized MnMgAl-LDH/IO NPs were synthesized via a facile hybridization strategy through conjugating positively charged MgMnAl-LDH NPs with negatively charged IO NPs (Figure 6A,B). Both T_1_ and T_2_ MRI enhancements were investigated in comparison with the MRI of MnMgAl-LDH NPs and IO NPs (Figure 6C). The r_1_ values of MnMgAl-LDH/IO NPs were 2-fold higher than those of MnMgAl-LDH NPs in terms of Mn (i.e., 4.46 mM^−1^ s^−1^ for MnMgAl-LDH/IO NPs vs. 2.14 mM^−1^ s^−1^ for MnMgAl-LDH NPs.). And the r_2_ values of MnMgAl-LDH/IO NPs were also higher than those of IO NPs in terms of Fe (i.e., 301.88 mM^−1^ s^−1^ for MnMgAl-LDH/IO NPs vs. 274.33 mM^−1^ s^−1^ for IO NPs.). This evidently enhanced T_1_ and T_2_ MRI contrast effect of MnMgAl-LDH/IO NPs could be resulted from the increased local magnetic field intensity which was due to the adsorption of IO NPs on the surface of MnMgAl-LDH NPs. Further in vivo MR imaging of tumor tissues in a tumor xenograft model revealed the significantly increased signals after the injection of as prepared NPs (Figure 6D).

#### 3.2.2. LDH Nanohybrids for MRI with Therapeutic Functions

**Figure 7:**
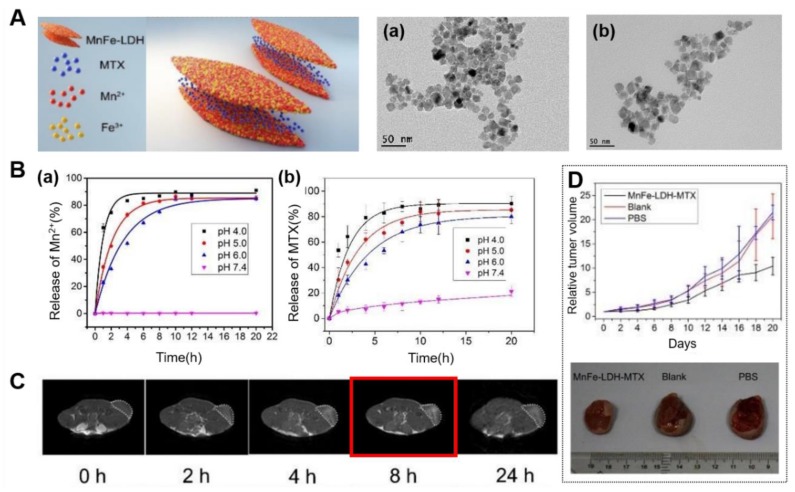
(**A**) Schematic illustration of MnFe-LDH-MTX, TEM images of MnFe-LDH (**a**) and MnFe-LDH-MTX (**b**), (**B**) release profiles of Mn ions from MnFe-LDH (**a**) and MTX from MnFe-LDH-MTX (**b**) in different pH buffers (n = 3), (**C**) in vivo T_1_-weighted MR images of S180 tumor bearing BALB/c nude mice after the administration of MnFe-LDH-MTX within 24 h, (**D**) tumor volume change of S180 tumor bearing mice during treatment and photo images of excised tumors collected at 20 days after treatment, reproduced from [11], with permission from the Royal Society of Chemistry, 2017.

In 2017, Yang et al. reported the MnFe-LDH for the first time, where Mn and Fe are essential trace elements for mammals and suitable for constructing LDH host layer for biomedical applications [11]. And they demonstrated pH-responsive T_1_ MRI contrast enhancement and the capability drug delivery with negatively charged chemotherapeutic drug MTX intercalated into the gallery of MnFe-LDH (Figure 7). The MnFe-LDH-MTX was synthesized by an in situ co-precipitation method and exhibited no significant changes on a platelet-like morphology (Figure 7A). The great enhancement in T_1_ relaxivity in acidic microenvironment of tumor was realized by releasing paramagnetic Mn^2+^ and Fe^3+^ ions from MnFe-LDH. The in vitro study with HeLa cells showed a great enhancement in T_1_ signals in the cells with the elapse of time, demonstrating that in acidic environment of endosomes/lysosomes, MnFe-LDH could be triggered to release Mn^2+^/Fe^3+^ ions, therefore leading to achieve brighter T_1_-weighted images (Figure 7B(a)). As shown in Figure 7B(b), change in the release amount of MTX under different pH conditions was measured to investigate the drug release behavior. The release at pH 7.4 was slow and only 21% MTX was released from MnFe-LDH within 20 h, while approximately 80%, 85%, and 90% were released at pH 6.0, 5.0, and 4.0, respectively. The comparison result showed that it could significantly promote the release of drug in the acidic environment. Moreover, they also demonstrated the excellent T_1_ contrast, effective drug delivery and related therapeutic abilities in a pH-controlled manner (e.g., in tumor area) through in vivo study.

### 3.3. Multimodal Imaging

As described above, researches in the bio-imaging fields based on nanomaterials has exploded and has shown the potential exists for next-generation biomedical technologies during the past few years. However, a single imaging modality alone cannot possess all the necessary capabilities for comprehensive imaging. Therefore, multimodal imaging strategy, in which the advantages of each imaging modality can be synergistically combined altogether and the disadvantages can be complemented by each other, is quickly becoming a quite useful tool to ultimately provide more accurate information [10,30,31,32,33,34,40,41,42,43,44,45]. Various combinations of different modalities are possible and in the following section, we will discuss some recent progress in synergistically integrated nanoparticle probes for CT/MR and fluorescence/MR dual modal imaging.

**Figure 8:**
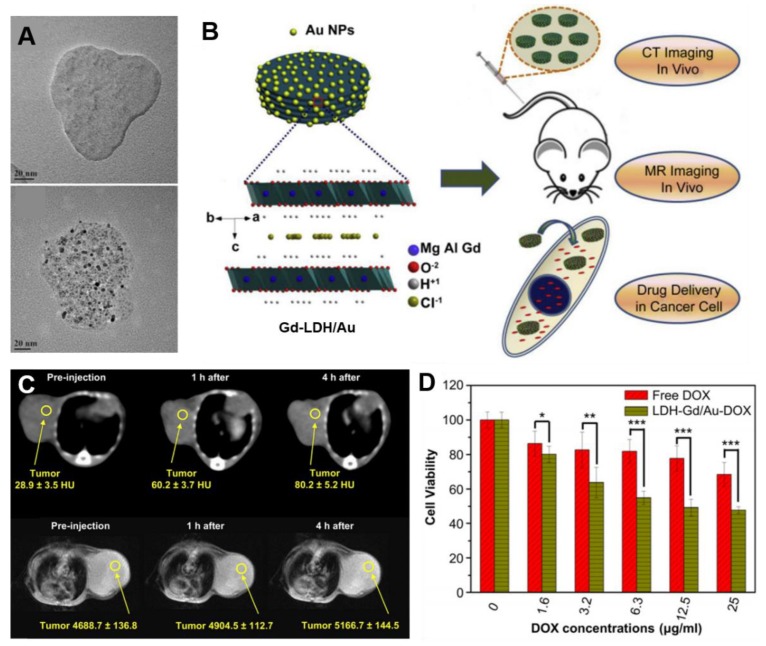
(**A**) TEM images of Gd-LDH and Gd-LDH/Au, (**B**) Schematic illustration of the Gd-LDH/Au as a theranostics platform for dual modal CT-MR imaging and anti-cancer drug delivery, (**C**) CT (top) and T_1_-weighted MR images (bottom) of tumor after intravenous injection of Gd-LDH/Au-heparin in 4 T1 murine breast tumor-bearing mice for 0 h, 1 h and 4 h; (**D**) HeLa cell viabilities when exposed to free DOX and Gd-LDH/Au-DOX at different concentrations (* *p* < 0.05, ** *p* < 0.01, *** *p* < 0.001), reproduced from [10], with permission from Elsevier Ltd., 2013.

For example, CT/MR dual-modal probes were developed by Shi et al. in 2013 [10] and it has been demonstrated that the Gd-doped MgAl-LDH/Au nanocomposite was a practically applicable platform as a diagnostic agent for bimodal imaging and a drug carrier monitored by non-invasive visualization both in vitro and in vivo (Figure 8B). In this study, a disk-shaped Gd-LDH/Au nanocomposite with a nanoparticle size of ≈138 nm was produced by functionalizing Au nanoparticles as CT contrast agent with a size of ≈3.4 nm on the surface of Gd-LDH (Figure 8A). The Gd-LDH/Au nanohybrids have been observed to be better contrast agents not only in vitro than the commercial agent Iobitridol and Magnevist, but also effective contrast agents in vivo for CT and T_1_-weighted MRI dual imaging. After surface modification prepared nanohybrids with heparin, they also showed effective in vivo CT and MR imagings of tumors after intravenous administration in 4T1 murine breast tumor-bearing mice. As shown in Figure 8D, the CT signal intensity value increased from 28.9 to 80.2 and T_1_-weighted MR value increased from 4688.7 to 5166.7 in 4 h after the intravenous administration, which demonstrated excellent in vivo targeting function through ERP effect and uptaking by cancer cells of Gd-LDH/Au nanohybrids. In addition, the loaded DOX was transported by Gd-LDH/Au via endocytosis into the cancer cell followed by pH-responsive release in the acidic cytoplasm and showed stronger cytotoxicity against cancer cells (Figure 8D).

**Figure 9:**
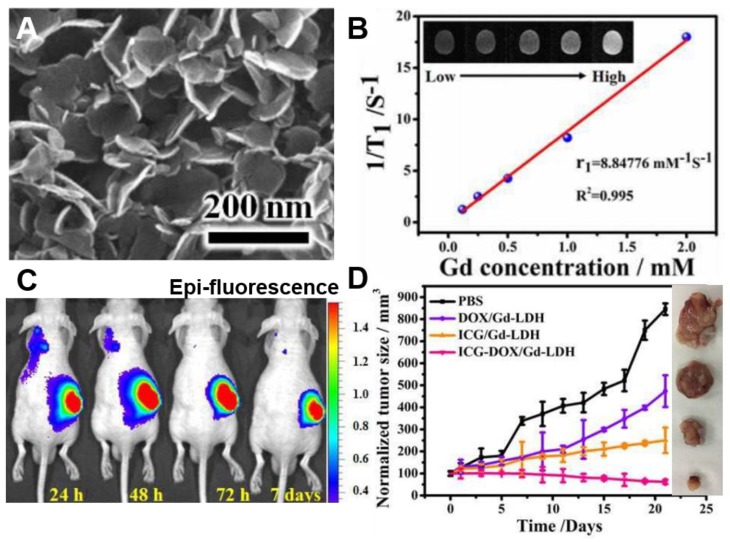
(**A**) SEM image of Gd-LDH/ICG-DOX, (**B**) analysis of relaxation rate 1/T_1_ vs. Gd concentration and corresponding T_1_-weighted MR images (inset), (**C**) in vivo fluorescence imaging Hela tumor bearing nude mice at 24, 48, 72 h, and seven days after intratumor injection of Gd-LDH/ICG-DOX, (**D**) the tumor growth curves under different treatments and photo images of excised Hela tumors collected at 20 days after corresponding treatment, reproduced from [30], with permission from the Royal Society of Chemistry, 2017.

One of the recent studies of progress in the field is utilizing a layered supramolecular nanovehicle, denoted as Gd-LDH/indocyanine green (ICG)-DOX, for fluorescence/MR dual modal imaging and synergistic cancer therapy [30]. They synthesized multifunctional nano-system, i.e., Gd-LDH/ICG-DOX by doping Gd^3+^ into the MgAl-LDH host layers, followed by intercalating both of DOX, the anti-tumor chemotherapeutic drug and ICG, the photothermal reagent into the Gd-LDH galley. As revealed in Figure 9A, the morphology of as-prepared material was a plate-like particle with a size of ≈120 nm with homogeneous dispersed and well stabilized DOX and ICG guest molecules into the interlayer of the LDH carrier. The doped Gd^3+^ and intercalated ICG could provide fluorescence/T_1_-weighted MR dual-modal imaging with great accuracy and sensitivity, which led to great contribution on the trace of drug distribution and tumor treatment (Figure 9B,C). The unique structure of LDH can stabilize the ICG-DOX and Gd-LDH/ICG-DOX could generate hyperthermia and reactive oxygen species (ROS) to enhance the photothermal therapeutic efficiency as well as the loading capacity of DOX under an irradiation in near infrared (NIR) range. The excellent chemo- and photothermal-combination therapy effects on tumor growth inhibition were also demonstrated through both in vitro studies with Hela cells and in vivo studies using Hela tumor bearing nude mice model (Figure 9D). Compared with both the Gd-LDH/DOX for chemo-therapy and Gd-LDH/ICG for photothermal therapy (PTT) groups, Gd-LDH/ICG-DOX for both chemo- and PTT have shown the greatest therapeutic ability. These phenomena imply the reciprocal promoting properties of DOX and ICG, for instance, the acceleration of the DOX release from LDH along with increase of temperature caused by ICG and the enhancement of the ^1^O_2_ generation possibly by forming non-covalent interactions between the DOX and ICG molecules. Despite the mechanism for such combinations needs further identification, the Gd-LDH/ICG-DOX was indicated to be a promising multifunctional platform for biomedical applications.

**Figure 10:**
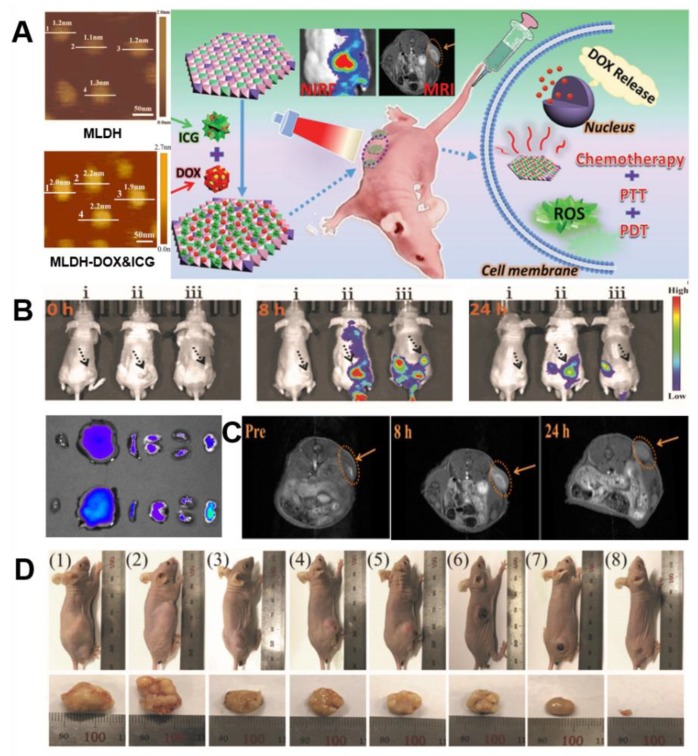
(**A**) AFM images of Gd-MLDH and Gd-MLDH-DOX&ICG nanosheets (left), schematic illustration for MLDH-based versatile platform that can be utilized for dual modal imaging including NIRF and MR imaging with the synergistic chemo-/PT/PD combination therapy (right). (**B**) In vivo fluorescence imaging of nude mice bearing HepG2 tumors at different time points after i.v. injection of i: saline, ii: MLDH-DOX&ICG, and iii: DOX&ICG (tumors are pointed out by the black arrows) and NIRF images of tumor and different organs after i.v. injection of DOX&ICG (top) and MLDH-DOX&ICG (bottom) at 24 h (respectively heart, liver, spleen, lung, kidney, tumor, from left to right). (**C**) In vivo T_1_-weighted MR images at different time points after i.v. injection of MLDH-DOX&ICG (tumor locations are indicated by the orange arrows). (**D**) Digital photographs of the mice on day 14 after various treatments and corresponding excised tumors (respectively from (1) to (8): saline, MLDH nanosheets, DOX&ICG, MLDH-DOX&ICG, MLDH-DOX with irradiation, DOX&ICG with irradiation, MLDH-ICG with irradiation, and MLDH-DOX&ICG with irradiation), reproduced from [57], with permission from WILEY-VCH Verlag GmbH & Co. KGaA, 2018.

More recently, Duan et al. reported ultrathin monolayered-double-hydroxide (MLDH) nanosheets as a versatile platform that can be utilized for dual modal imaging including near infrared fluorescence (NIRF) and MR imaging and a highly desirable for multimodal image-guided combination therapy, which showed the synergistic chemo/PTT/PDT effect of cancer treatment [57]. Gd^3+^-doped MLDH/DOX-ICG nanosheets were prepared via a new “bottom-up” strategy, while the previously reported ultrathin 2D structured nanosheets were mainly synthesized based on a “top-down” method [82,83,84,85]. This LDH-based multifunctional system could be endowed with a precisely controlled chemical composition, uniform morphology/size, and especially high specific surface area (Figure 10A), which resulted in an optimal drug loading content of 797.36% and an encapsulation efficiency of 99.67% for DOX and ICG. The ultrahigh drug loading has been explained by virtue of two unique properties of reported MLDH: (1) ultrathin nanosheets with an ultrahigh surface area which indicate that a lateral size of ≈70 nm with a thickness of ≈1.2 nm for pristine MLDH nanosheets and a lateral size of ≈80 nm with an increased thickness of ≈2.0 nm after co-loading of DOX and ICG; (2) the combined electrostatic interaction between DOX, ICG and MLDH nanosheets may owe to primary amine group in DOX molecule and sulfonate group in ICG molecule.

It was confirmed that diagnosis for tumor can be performed noninvasively using in vivo dual imaging technique including NIRF and MR imaging. The T_1_-weighted relaxivity r_1_ value, which is a key parameter for evaluating the contrast agent, was determined to be 7.93 × 10^−3^ M^−1^s^−1^ in Gd-MLDH/DOX-ICG and this was found to be about 2.27-fold larger than the r_1_ value of the conventional Gd-DTPA of 3.5 × 10^−3^M^−1^s^−1^. In order to observe the in vivo biodistribution and tumor accumulation of Gd-MLDH/DOX-ICG, nude mice bearing HepG2 tumors were investigated through both NIRF and MR imaging. After intravenous (i.v.) injection of MLDH/DOX-ICG in mice, T_1_-enhanced MR signal at the tumor site gradually increased, showed the strongest intensity after 8 h and the effect continued for 24 h (Figure 10C). In accordance with the MRI results, the fluorescence signal was observed after 2 h, the strongest signal was presented at 8 h and a significant intensity was maintained till 24 h. The ex vivo fluorescence imaging for the tumor and other normal organs at 24 h post administration showed the strongest fluorescence signal at the tumor site (Figure 10B). As is studied, NIRF/MRI dual imaging was able to provide clear information on enhanced uptake and effective accumulation in the tumor area of Gd-MLDH/DOX-ICG nanocomposite material with excellent penetration, high spatial resolution and real-time visualization.

In addition, from both in vitro and in vivo studies, the as-prepared Gd-MLDH/DOX-ICG revealed a chemo-/PT/PD trimode combination therapy which originates from accurate delivery and release of DOX anticancer drugs by pH controlled and NIR-irradiation, as well as significantly large ROS production. As showed in Figure 10D, notably, the treatment with Gd-MLDH/DOX-ICG upon irradiation exhibited the strongest ability toward tumor elimination, indicating a remarkably enhanced synergistic anticancer therapeutic effect. Furthermore, the superior biocompatibility of Gd-MLDH/DOX-ICG was proved as well.

## 4. Conclusions

A number of strategic properties of LDHs, such as biocompatibility, unique host–guest interactions, surface functionality, tunable particle size, high loading capacity of biomolecules, protection of drugs or genes within interlayer galleys, pH-responsive solubility and sustained release, can contribute to the cellular uptake, circulation, distribution and clearance behaviors in vivo. The recently emerged LDH nanomaterials for bio-imaging applications with therapeutic functions are briefly summarized in Table 1. In this review, the features and synthesis strategies of nanoparticle probes for bio-imaging based on LDH are summarized and functional LDH materials for bio-imaging, which are being developed focusing on fluorescence imaging, MRI and multimodal imaging, i.e., CT/MRI and fluorescence imaging/MRI, have been specifically described. Furthermore, we highlighted recent in vitro and in vivo results, which have confirmed their potential as enhanced bio-imaging agents as well as drug delivery vehicles.

As the medical paradigm is changing from the treatment of disease to the early diagnosis and prevention, the need for promptness and reliability improvement in the field of diagnosis is rising. The development of a novel high sensitivity nano contrast agent system for molecular bio-imaging, being combined with drug delivery, gene therapy, and photothermal therapy and so on, is expected to monitor the treatment process of diseases by real-time imaging, realize early diagnosis of diseases accurately, and provide a platform to develop customized therapies or to accelerate the development of new drugs. With further improvement in noninvasive bio-imaging techniques, the LDH-supported imaging agent systems are expected to contribute more to clinical applications in the future.

## Figures and Tables

**Table 1:** A brief summary of LDH nanomaterials for biomedical imaging applications.

LDH Host	Contrast Agents	Therapeutic Agents	Molecular Engineering	Applications	Key Feature	References
MgAl	FITC	siRNA	Silane coupling, self-assembly, size control	Fluorescence imaging, gene-therapy	Selective tumor targeting conjugated with FA, siRNA-based gene-therapy in vitro and in vivo	[6]
MgAl	FITC	MTX	Intercalation (coprecipitation and ion exchange), size control	Fluorescence imaging, chemo-therapy	Intercellular uptake mechanism: clathrin-mediated endocytosis	[9]
MgAl	ICG		Intercalation, covalent coating	NIRF imaging	Organ-specific drug delivery system	[20]
MgAl	FITC		Silane coupling, size control	Fluorescence imaging	Intracellular fate and trafficking mechanism: endolysosomal escape for 100 nm nanoparticles	[21]
MgAl	FITC	DNA, adenosine triphosphate	Intercalation (ion exchange)	Fluorescence imaging, gene-therapy	Gene delivery system with high transfection efficiency	[22]
MgAl	Cy5	DOX	A base-catalyzed coprecipitation	Fluorescence imaging, chemo-therapy	Internalization into cancer cells mechanism: macropinocytosis, clathrin- and lipid raft/caveolae-mediated endocytosis	[23]
MgAl	CDs, ICG	ICG	Self-assembly, ultrathin LDHs	Fluorescence imaging, photoacoustic imaging, two-photon imaging, PTT	Multifunctional theranostic nanocarrier system for the cancer treatment	[34]
MgAl	FITC		Silane coupling, size control	Fluorescence imaging	Targeted cellular uptake mechanism: particle size dependant clathrin-mediated endocytosis	[35]
MgAl	FITC, ICG	ICG	Intercalation (ion exchange), covalent coating	Fluorescence imaging, PDT	High photo-toxicity of PDT due to the enhanced protection against photo and thermal degradations	[53]
MgAl	Cy7, FITC		Self-assembly	Fluorescence imaging, brain targeting	Enhanced brain cell targeting and cellular transportation for efficient brain disease treatment (ligand-modified LDH)	[25]
GdMgAl	Gd^3+^, Au NPs	DOX	Substitution, self-assembly	MRI, CT, chemo-therapy	Selective cancer targeting in vivo through EPR effect	[10]
GdMgAl	Gd^3+^, ICG	DOX, ICG	Co-intercalation	MRI, fluorescence imaging, chemo-therapy, PTT	Multifunctional theranostic nano-systems for the cancer treatment	[30]
GdMgAl	Gd^3+^, ICG	DOX, ICG	MLDH, a novel bottom-up method	MRI, NIRF imaging, chemo-therapy, PTT, PDT	An ultrahigh drug loading content (LC): 797.36%, an encapsulation efficiency (EE): 99.67%	[57]
MnMgAl	Mn^2+^		Coprecipitation, isomorphic substitution	MRI	pH-ultrasensitive T_1_-MRI performance (even with pH 6.5–7.0, i.e., the pH range in a tumor microenvironment)	[26]
MnMgAl	Mn^2+^, IO NPs		Coprecipitation, isomorphic substitution, self-assembly	MRI (T_1_/T_2_)	Enhanced T_1_/T_2_ MRI signals both in vitro and in vivo	[12]
MnAl	Mn^2+^	siRNA	Coprecipitation, self-assembly	MRI, gene-therapy	An effective anticancer drug/gene delivery system, T_1_-weighted MRI in brain cancer theranostics	[60]
MnFe	Mn^2+^	MTX	Coprecipitation,	MRI, chemo-therapy	The first work on MnFe-LDH	[11]
ZnAl	Gd-DTPA		Coprecipitation, size control	MRI	Similar T_1_-weighted MR contrast effect, a suitable particle size for in vivo	[27]
DyZnAl	Dy^3+^	Folate, ibuprofen and gallate ions	Coprecipitation, intercalation	MRI, drug delivery system	Theranostic materials with luminescent and magnetic properties	[54]
